# Exploring the pathways between personality traits, alexithymia, and resilience among medical students and interns: a cross-sectional study in Egypt

**DOI:** 10.1038/s41598-026-59508-5

**Published:** 2026-07-02

**Authors:** Hajer Azzam, Yusof Mohamed Omar, Kariem Awad, Ahmed H. Ata, Mennatullah Elagouz, Mariam H. Nabih, Mahmoud ElKaffas, Mohamad A. Helaiel, R. P. A. Team of Collaborators, Mohamed Elmekkawi, Rahma M. Almetwaly, Samaa T. Hassan, Nada E. Mokhtar, Omar Reisha, Hesham Helmy, Ibtihal M. A. Ibrahim

**Affiliations:** 1 Faculty of Medicine, Mansoura University, Mansoura, Egypt; 2 AZ Zurayqi Health Unit, El Senbellawein City, Dakahlia, Ministry of Health, Dakahlia, Egypt; 3 Psychiatry department, Faculty of Medicine, Mansoura University, Mansoura, Egypt; 4 Mansoura Manchester Research Society (MMRS), Faculty of Medicine, Mansoura, Egypt

**Keywords:** Personality traits, Alexithymia, Resilience, Medical students, Egypt, Psychology, Medical research

## Abstract

Resilience is essential for medical students, yet how personality traits are associated with resilience through alexithymia remains underexplored. This cross-sectional study, conducted from December 2023 to May 2024 using printed surveys and convenience sampling, included 598 participants (412 medical students and 186 interns). The survey included the Connor-Davidson Resilience Scale (CD-RISC-10), the Toronto Alexithymia Scale (TAS-20), and the NEO Five-Factor Inventory (NEO-FFI). Pearson’s correlation (*r*), multivariable linear regression, and path analysis were conducted to examine factors associated with resilience. Resilience was negatively correlated with neuroticism and alexithymia (*r* = -0.41 and -0.32, *p* < 0.001) and positively correlated with extraversion and conscientiousness (*r* = 0.29 and 0.36, *p* < 0.001). Regression identified neuroticism, externally-oriented thinking, agreeableness, and current psychiatric disorder as negative predictors (β = -0.32, -0.14, -0.10, and -0.45 respectively, p ≤ 0.012), while extraversion and conscientiousness were positive predictors (β = 0.14 and 0.24 respectively, p < 0.001). On path analysis, externally-oriented thinking provided a significant indirect statistical link in the associations of conscientiousness (β = 0.02, p = 0.019) and openness (β = 0.03, p = 0.010) with resilience. Addressing neuroticism and alexithymia subcomponents may enhance resilience and support student well-being.

**Supplementary Information:**

The online version contains supplementary material available at 10.1038/s41598-026-59508-5.

## Introduction

Medical students are a particularly vulnerable population, with studies consistently reporting higher rates of psychological distress and mental health issues amongst them compared to their non-medical peers^1,2^. This vulnerability is commonly attributed to the demanding nature of medical education, including heavy academic workloads, frequent examinations, and persistent performance pressure. At the same time, individual differences in emotional processing and personality may shape how students perceive, regulate, and adapt to these stressors. Difficulties in identifying and managing emotions^3,4^, alongside maladaptive personality traits^5^, may impair students’ ability to cope effectively with adversity, thereby increasing their susceptibility to burnout, anxiety, depression, and other forms of psychological distress. Left unaddressed, these mental health concerns can adversely affect students’ well-being, academic performance, professional development, and ultimately the quality of patient care they provide in the future^6^.

Nevertheless, understanding how students adapt to the demands of medical education requires distinguishing between relatively stable personality traits and more dynamic adaptive capacities such as resilience. The Five-Factor Model conceptualizes personality through broad enduring dimensions—including neuroticism, extraversion, conscientiousness, agreeableness, and openness to experience—that influence emotional, cognitive, and behavioral responses to stressors^7,8^. Resilience, in contrast, refers to a dynamic process and capacity through which individuals adapt positively and recover from adversity or stress^9^. Although resilience is influenced by environmental and contextual factors^10^, personality traits are thought to provide an important dispositional foundation for resilient functioning^11^.

For instance, among these personality traits, neuroticism has consistently been associated with lower resilience, likely due to heightened emotional reactivity, greater perceived stress, and a tendency toward maladaptive coping strategies^12,13^. Conversely, traits such as extraversion and conscientiousness have been associated with higher resilience, which is believed to be due to their role in facilitating social connectedness, effective self-regulation, and greater utilization of support systems^14^. Thus, personality traits may influence not only how stress is experienced, but also the psychological resources available to manage it. Investigating these relationships is particularly relevant in the Egyptian medical education context, where studies have reported high levels of maladaptive personality traits^15,16^, reluctance to seek psychological support^17,18^, and generally low resilience among medical students^19,20^.

Beyond the direct association between personality traits and resilience, alexithymia may represent an important intermediary variable underlying this relationship. Alexithymia is a cognitive-affective construct characterized by difficulties identifying emotions (DIF), difficulties describing emotions to others (DDF), and an externally oriented thinking style (EOT)^21^. While alexithymia demonstrates relative trait stability^22^, it differs conceptually from broad personality dimensions by reflecting a more specific impairment in emotional awareness and emotional processing. Such impairments may interfere with adaptive coping and emotional regulation, both of which are central to resilient functioning. For example, even students with protective personality traits such as conscientiousness or extraversion may struggle to effectively utilize social support or regulate stress if they are unable to recognize and articulate their emotional experiences^23^. In this way, alexithymia may partially explain how underlying personality traits translate into either adaptive or maladaptive responses to stress.

This relationship is particularly concerning within medical education, where students are repeatedly exposed to emotionally demanding situations while simultaneously operating in highly competitive academic environments^24^. Students with both high alexithymic traits and low resilience may therefore be at increased risk of developing psychiatric disorders, severe psychological distress, and suicidal ideation^25^. Furthermore, studies among Egyptian medical students consistently report substantial hesitancy toward seeking mental health support, with the most commonly cited barrier being a preference to manage problems independently^18^. Although not synonymous with alexithymia, this pattern may reflect underlying difficulties in emotional recognition, expression, or help-seeking behaviors, further highlighting the potential relevance of alexithymia within this population.

Nevertheless, despite growing evidence demonstrating the prevalence of maladaptive personality traits^15,16^, emotional processing difficulties^17,18^, and low resilience among medical students in Egypt^19^, the pathways potentially linking these constructs remain insufficiently studied. Most existing studies have examined these variables independently, with limited investigation into how personality traits may be indirectly associated with resilience through emotional processing pathways such as alexithymia. Addressing this gap may provide a more comprehensive understanding of vulnerability and adaptation among medical students, while also informing targeted psychological interventions aimed at strengthening resilience and emotional coping.

Accordingly, the present study adopts a conceptual framework grounded in theoretical models of personality-based stress adaptation^26^. Within this framework, personality traits (e.g., neuroticism and conscientiousness) are conceptualized as foundational predispositions influencing stress perception and coping tendencies, whereas alexithymia is positioned as a secondary cognitive-affective processing construct that may explain the indirect associations between these traits and resilience. Therefore, this study aimed to: (1) quantify resilience and its demographic correlates among medical students and interns at Mansoura University, (2) identify the independent predictors of resilience after controlling for demographic variables, and (3) test a path model evaluating whether specific alexithymia subcomponents serve as an indirect statistical link in the relationship between personality traits and resilience.

## Methods

### Study design and setting

A cross-sectional study with descriptive and analytical components was conducted at the Faculty of Medicine, Mansoura University. Data were collected between December 2023 and May 2024. The study received ethical approval from the Institutional Research Board (IRB) at Faculty of Medicine, Mansoura University (R.23.11.2394). At the beginning of the survey, a written informed consent was obtained from participants. Participants took part voluntarily, and were ensured anonymity and confidentiality of their responses.

### Sample size calculation

The required sample size was calculated using the OpenEpi online calculator^27^, with a 50.06% prevalence of low resilience reported in a multicentric study among Egyptian medical students^19^, a population size of 10,443, a 95% confidence interval, and a 5% margin of error. The minimum required sample size was determined to be 371 participants. However, 620 printed surveys were distributed and a total of 598 complete responses were obtained, for a 96% response rate, thus exceeding the minimum required sample size and ensuring adequate representation of the population.

### Study population

The study sample included first to fifth year medical students and interns enrolled in the three programs of the faculty: Mansoura Manchester Program for Medical Education (MMPME), Integrated, and Conventional programs. The Integrated program is the primary government-funded track, initiated following Egypt’s recent medical education reform that emphasized competency-based education^28^. However, at the time of conducting the study, there were two remaining classes of the old conventional program pre-reform. The MMPME, conversely, is a self-financed program offered in collaboration with the University of Manchester. In this track, students benefit from high tutor engagement, small working groups, and problem-based learning.

### Data collection approach

Printed surveys were used to collect data in lecture halls, clinical rounds, and the faculty grounds. Inclusion criteria were undergraduate medical students from the first to fifth academic years and interns; enrollment in the Faculty of Medicine, Mansoura University; and affiliation with any of the three educational programs (MMPME, Integrated, or Conventional). Exclusion criteria included refusal to provide informed consent and submission of incomplete questionnaires with missing data, which were removed from the final analysis.

### Data collection tools

The survey comprised three English questionnaires alongside a section collecting socio-demographic data, including gender, nationality, age, academic year, faculty program, and present, past, and family history of psychiatric disorders diagnosed by a professional.

Resilience was assessed using the 10-item Connor-Davidson Resilience Scale (CD-RISC-10)^29^. The CD-RISC-10 is a valid scale that evaluates an individual’s ability to cope with challenges such as change, personal issues, illness, stress, failure, and emotional distress^29^. The scale is scored using 5-point Likert scales ranging from 0 (never) to 4 (almost always), with higher scores indicating higher resilience. In the development study, the CD-RISC-10 demonstrated good internal consistency (α = 0.85) and construct validity, with a robust unidimensional factor structure confirmed via exploratory and confirmatory factor analyses. In this study, students were classified as “low” and “high” resilience according to the validated cutoff of 25.5 proposed by Ye et al. to help physicians and nurses quickly identify individuals at high risk of psychological distress^30^.

Alexithymia was measured using the 20-item Toronto Alexithymia Scale (TAS-20)^31,32^, a self-report instrument assessing difficulties in identifying and describing emotions. The scale demonstrates a three-factor structure corresponding to its three subscales: DIF (7 items), DDF (5 items), and EOT (8 items). Responses to the TAS-20 are rated on a 5-point Likert scale (1 = strongly disagree to 5 = strongly agree). The scale has demonstrated good internal consistency in its original validation (α = 0.81) as well as good test–retest reliability over a 3-week interval (*r* = 0.77)^33^. Scores classify individuals as having no alexithymia (0–51), possible alexithymia (52–60), or alexithymia (61–100)^31,32^. These cutoffs have been used frequently in previous studies, including studies involving Egyptian^34^ and other adult Arab student populations^35,36^.

Personality traits were evaluated using the NEO Five-Factor Inventory (NEO-FFI)^33^. The NEO-FFI is a 60-item self-report questionnaire that provides a reliable measure of the five major personality domains. These domains include: Neuroticism, Extraversion, Openness, Agreeableness, and Conscientiousness, with 12 items dedicated to assessing each trait. Responses to the NEO-FFI are rated on a 5-point Likert scale (1 = strongly disagree to 5 = strongly agree). Previous psychometric evaluation has supported the factor structure and reliability of the inventory, with internal consistency coefficients ranging from 0.68 to 0.86 across the subscales^33^. Additionally, convergent validity has been established through substantial cross-observer agreement between self-reports and peer ratings (correlations ranging from 0.25 to 0.62)^33^.

### Statistical analysis

Jamovi statistical software was used for statistical analysis. Categorical variables were summarized as frequencies and percentages. Normality was assessed using the Shapiro–Wilk test and visual inspection of Quantile–Quantile (Q-Q) plots. Although the Shapiro–Wilk test is highly sensitive in large samples, an inspection of skewness and kurtosis confirmed the normal distribution of the data; thus, continuous variables were reported as means and standard deviations (SDs) and parametric tests were utilized. Independent-samples t-tests were used to compare mean resilience scores between two groups, and One-way ANOVA was applied to compare mean resilience scores across variables with more than two categories. Pearson’s correlation coefficient was used to assess relationships between continuous variables.

To identify independent predictors of resilience, a multivariable linear regression analysis was conducted using the ‘enter’ method., incorporating sociodemographic (e.g., age, sex, program) and psychological variables (alexithymia, personality traits). Categorical variables were entered into the regression model as dummy-coded variables. Prior to interpretation, regression assumptions were assessed, including multicollinearity using the Variance Inflation Factor (VIF). Subsequently, a path analysis utilizing Maximum Likelihood (ML) estimation was performed in an exploratory capacity to examine the direct and indirect effects of personality traits on resilience through alexithymia subcomponents. The model was specified with the five personality traits (neuroticism, extraversion, openness, agreeableness, and conscientiousness) entered as exogenous variables, the three alexithymia subcomponents (difficulty identifying feelings, difficulty describing feelings, and externally-oriented thinking) as mediators, and resilience as the endogenous variable. Because all possible direct and indirect pathways between the included variables were estimated, the model was fully saturated; therefore, traditional model fit indices were not applicable. Furthermore, because indirect effects often violate the assumption of normality, their significance and 95% Confidence Intervals (CIs) were estimated using a bias-corrected bootstrapping procedure with 5,000 resamples. Internal reliability of the utilized scales was examined using Cronbach’s alpha. For all analyses, a p-value of < 0.05 was considered statistically significant. Questionnaires with missing data were excluded from the analysis.

## Results

### Sample characteristics and descriptive statistics

The study included 412 medical students (68.9%) and 186 interns (31.1%). The majority were female (55.9%), Egyptian (72.7%), resided in urban areas (75.1%), and were single (97%). A past psychiatric disorder diagnosed by a professional was reported by 4.5% of students, while 9.4% had a family history of psychiatric disorders (Table 1).

**Table 1:** Study participants’ characteristics and differences in resilience scores (*N* = 598).

Variable		n (%)	Resilience score (Mean (SD))	p-value
Sex	Female	334 (55.9%)	24.2 (5.3)	0.001
	Male	264 (44.1%)	25.9 (6.3)	
Academic year	1 st year	72 (12%)	24.2 (6.6)	0.8
	2nd year	83 (13.9%)	25.3 (5.1)	
	3rd year	74 (12.4%)	24.8 (4.6)	
	4th year	98 (16.4%)	25.5 (6)	
	5th year	85 (14.2%)	24.7 (5.8)	
	Intern	186 (31.1%)	24.9 (6.2)	
Program	Conventional	343 (57.4%)	26 (7)	
	Integrated	201 (33.6%)	24.4 (5.6)	0.04
	MMPME	54 (9%)	25.5 (5.8)	
Nationality	Egyptian	435 (72.7%)	24.7 (5.7)	0.22
	Foreign	163 (27.3%)	25.4 (6.1)	
Residence	Urban	449 (75.1%)	24.9 (6)	0.85
	Rural	142 (23.7%)	25 (5.2)	
	Other	7 (1.2%)	23.7 (4.2)	
Marital status	Single	580 (97%)	25 (5.8)	0.55
	Not single	18 (3%)	24.1 (5.3)	
Present psychiatric disorder	No	573 (95.8%)	25 (5.8)	0.02
	Yes	25 (4.2%)	22.2 (5)	
Past psychiatric disorder	No	571 (95.5%)	25 (5.8)	0.57
	Yes	27 (4.5%)	24.3 (6.5)	
Family history of psychiatric disorder	No	542 (90.6%)	25 (5.7)	0.28
	Yes	56 (9.4%)	24.1 (6.6)	
Alexithymia	No	271 (45.3%)	26.5 (5.7)	< 0.001
	Possible	176 (29.4%)	24.1 (5.5)	
	Yes	151 (25.3%)	22.9 (5.6)	

Table 2 summarizes the descriptive statistics and internal consistency reliabilities of the study variables. The mean resilience score for the study population was 24.9 (SD = 5.8), with 50.8% having low resilience. The CD-RISC-10 and TAS-20 total scores demonstrated acceptable reliability (α = 0.725 and 0.773, respectively). Among TAS-20 subscales, DIF showed acceptable reliability (α = 0.793), whereas DDF (α = 0.688) and EOT (α = 0.460) showed lower reliability. For the NEO-FFI traits, Neuroticism and Conscientiousness demonstrated acceptable reliability (α > 0.70), while Extraversion, Agreeableness, and particularly Openness showed relatively low internal consistency (α = 0.421–0.659).

**Table 2:** Descriptive statistics and internal consistency reliabilities for study variables.

Variable	Mean (SD)	Range	Cronbach’s α
Resilience (CD-RISC-10)	24.9 (5.83)	0–40	0.725
Alexithymia (TAS-20)			
Total score	53.1 (10.8)	20–100	0.773
Difficulty identifying feelings	18.1 (5.92)	7–35	0.793
Difficulty describing feelings	14.3 (4.21)	5–25	0.688
Externally-oriented thinking	20.7 (4.46)	8–40	0.46
Personality traits (NEO-FFI)			
Neuroticism	37.0 (7.40)	12–60	0.742
Extraversion	38.6 (5.95)	12–60	0.602
Openness	36.9 (5.31)	12–60	0.421
Agreeableness	42.1 (6.33)	12–60	0.659
Conscientiousness	41.7 (6.74)	12–60	0.743

### Bivariate associations and correlations

Males had significantly higher resilience scores than females (25.9 vs. 24.2, *p* = 0.001). Students in the conventional program had the highest resilience scores (26), followed by those in the MMPME (25.5) and integrated (24.4) programs (*p* = 0.04). Participants with a current psychiatric disorder had significantly lower resilience scores than those without (22.2 vs. 25, *p* = 0.02). Additionally, students with alexithymia exhibited significantly lower resilience than those without (22.9 vs. 26.5, *p* < 0.001) (Table 1).

Resilience was negatively correlated with neuroticism (*r* = −0.41, *p* < 0.001) and alexithymia total scores (*r* = −0.32, *p* < 0.001). Among the subcomponents of alexithymia, DIF, DDF, and EOT showed significant negative correlations with resilience (*r* = −0.19 to −0.20, *p* < 0.001). In contrast, resilience was positively correlated with extraversion (*r* = 0.29, *p* < 0.001) and conscientiousness (*r* = 0.36, *p* < 0.001) (Table 3).

**Table 3:** Correlation between resilience, alexithymia, and personality traits scores.

Variable	Correlation with resilience (*r*)	P-value
Alexithymia (TAS-20)		
Total score	−0.32	** < 0.001**
Difficulty identifying feelings	−0.19	** < 0.001**
Difficulty describing feelings	−0.19	** < 0.001**
Externally-oriented thinking	−0.20	** < 0.001**
Personality traits (NEO-FFI)		
Neuroticism	−0.41	** < 0.001**
Extraversion	0.29	** < 0.001**
Openness	0.02	0.58
Agreeableness	0.08	0.06
Conscientiousness	0.36	** < 0.001**

### Multivariable analysis and path modeling

In the multivariable linear regression model (Table 4), higher neuroticism (β = − 0.32, p < 0.001), EOT (β = − 0.14, p < 0.001), agreeableness (β = − 0.10, p = 0.01), and current psychiatric disorder (β = − 0.45, p = 0.012) were significant negative predictors of resilience. In contrast, extraversion (β = 0.14, p < 0.001) and conscientiousness (β = 0.24, p < 0.001) were significant positive predictors. All VIFs, except for age, ranged from 1.02 to 1.39, while age showed a VIF of 2.66. Tolerance values ranged from 0.376 to 0.976, indicating no evidence of problematic multicollinearity. The model explained 29% of the variance in resilience scores (Adjusted R2 = 0.294).

**Table 4:** Linear regression of factors associated with resilience among the study’s participants.

Variable	Category	Unstandardized B (95% CI)	Standardized β	p-value
Age	—	−0.11 (−0.57, 0.35)	−0.04	0.646
Gender	Female	Reference	—	—
	Male	0.25 (−0.66, 1.16)	0.04	0.592
Academic year	1 st year	Reference	—	—
	2nd year	1.11 (−0.56, 2.78)	0.19	0.192
	3rd year	0.82 (−1.11, 2.74)	0.14	0.404
	4th year	1.58 (−0.64, 3.81)	0.27	0.163
	5th year	1.07 (−1.45, 3.59)	0.18	0.405
	Intern	1.12 (−1.94, 4.18)	0.19	0.471
Program	Conventional	Reference	—	—
	Integrated	−1.52 (−3.21, 0.18)	−0.26	0.079
	MMPME	−0.81 (−2.48, 0.85)	−0.14	0.338
Nationality	Egyptian	Reference	—	—
	Non-Egyptian	0.79 (−0.20, 1.78)	0.14	0.12
Residence	Urban	Reference	—	—
	Rural	0.32 (−0.68, 1.32)	0.06	0.526
	Other	−2.32 (−6.07, 1.43)	−0.4	0.224
Marital status	Single	Reference	—	—
	Not single	−0.61 (−2.97, 1.75)	−0.1	0.614
Present history of psychiatric disorders	No	Reference	—	—
	Yes	−2.64 (−4.70, −0.58)	−0.45	**0.012**
Past history of psychiatric disorders	No	Reference	—	—
	Yes	0.54 (−1.56, 2.63)	0.09	0.616
Family history of psychiatric disorders	No	Reference	—	—
	Yes	−0.29 (−1.74, 1.15)	−0.05	0.692
Difficulty describing feelings	—	0.04 (−0.08, 0.17)	0.03	0.499
Difficulty identifying feelings	—	−0.06 (−0.15, 0.03)	−0.06	0.194
Externally-oriented thinking	—	−0.18 (−0.27, −0.08)	−0.14	** < 0.001**
Neuroticism	—	−0.25 (−0.31, −0.19)	−0.32	** < 0.001**
Extraversion	—	0.14 (0.06, 0.21)	0.14	** < 0.001**
Openness	—	0.05 (−0.03, 0.13)	0.04	0.247
Agreeableness	—	−0.09 (−0.16, −0.02)	−0.1	**0.01**
Conscientiousness	—	0.21 (0.15, 0.28)	0.24	** < 0.001**

To further evaluate exploratory indirect pathways, a bootstrapped path analysis was conducted. A summary of all significant direct and indirect pathways is presented in Table 5, while all tested pathways, including non-significant routes, are detailed in Supplementary Table 1. The path analysis showed that conscientiousness (β = 0.24, p < 0.001) and extraversion (β = 0.15, p < 0.001) maintained positive direct associations with resilience. Conversely, neuroticism (β = − 0.31, p < 0.001), agreeableness (β = − 0.11, p = 0.007), and EOT (β = − 0.12, p = 0.003) exhibited significant negative direct effects. Openness, DDF, and DIF were not significant direct predictors. Regarding indirect effects, EOT provided a significant indirect statistical link in the associations of conscientiousness (β = 0.02, p = 0.019) and openness (β = 0.03, p = 0.010) with resilience.

**Table 5:** Significant direct and indirect effects in the path model predicting resilience among the study’s participants.

Pathway	Unstandardized estimate B (95% CI)	Standardized β	p-value
Significant direct effects on resilience			
Neuroticism → Resilience	−0.24 (−0.31, −0.18)	−0.31	** < 0.001**
Extraversion → Resilience	0.15 (0.07, 0.23)	0.15	** < 0.001**
Agreeableness → Resilience	−0.10 (−0.17, −0.03)	−0.11	**0.007**
Conscientiousness → Resilience	0.21 (0.13, 0.29)	0.24	** < 0.001**
Externally-oriented thinking (EOT) → Resilience	−0.16 (−0.26, −0.05)	−0.12	**0.003**
Significant indirect effects on resilience			
Conscientiousness → EOT → Resilience	0.02 (0.01, 0.03)	0.02	**0.019**
Openness → EOT → Resilience	0.04 (0.01, 0.06)	0.03	**0.01**

## Discussion

Given the various challenges experienced by medical students throughout their education, understanding the factors that influence their resilience and ability to cope with these challenges is essential for graduating resilient future healthcare providers and designing effective support interventions. We found that resilience was significantly lower among females and students with a psychiatric disorder. Furthermore, our models demonstrated that lower resilience was strongly associated with high negative affectivity (neuroticism) and deficits in emotional processing (alexithymia), whereas extraversion and conscientiousness were significant positive predictors.

The mean resilience score of our study’s participants using the CD-RISC-10 was 25. This is notably lower than reported resilience scores among Chinese undergraduates (26.38)^37^, Korean medical students (27.06)^38^, and Mayo clinic residents (31.5)^39^. The significance of this finding becomes clearer when interpreted through the cutoff suggested by Ye et al.^30^, who proposed a threshold of 25.5 to help physicians and nurses quickly identify individuals at high risk of psychological distress. In line with this, a recent multicenter study among Egyptian medical students found that those with low resilience—who comprised 50.06% of the sample—were four times more likely to experience significant stress in multivariate analysis^19^. These findings suggest that medical students, both in our study and across Egypt, may be psychologically vulnerable due to lower resilience levels, underscoring the need to identify the underlying factors associated with resilience and develop strategies to strengthen it in this population.

In our study, male students exhibited significantly higher resilience scores than female students. This finding is consistent with previous research among medical students across different cultures and nations, including western and eastern cultures such as Egypt^19^, Canada^40^, and Germany^41^. Rather than stemming solely from intrinsic psychological differences, this disparity may be due to career-specific barriers faced by female students and doctors within Arab countries. These include a male-dominated clinical and academic hierarchy where women consistently report lower job satisfaction and are systematically excluded from senior leadership roles^42^. Consequently, these women confront an intense conflict between the demands of their chosen profession and the prevailing cultural expectation to prioritize marriage and domestic responsibilities^43^, all of which can result in greater stress and lower long-term resilience. Nevertheless, conflicting evidence exists, with some studies reporting no differences according to gender or even higher resilience among females^44–46^. These inconsistencies suggest that resilience is not solely determined by sex but rather further associated with various factors such as differences in mental well-being, personality traits, and emotional regulation capacity.

Indeed, in our study, students with an existing psychiatric condition and those exhibiting higher levels of neuroticism had significantly lower resilience scores. The link with neuroticism is especially notable, as this trait is marked by emotional instability and a tendency toward negative affect, both of which can undermine the emotional regulation needed for mental well-being in medical students within the demanding context of medical education^47,48^. Our path analysis further illuminated these relationships and found that while conscientiousness and extraversion positively predicted resilience, agreeableness and openness did not show significant positive direct associations. Notably, agreeableness emerged as a significant negative direct predictor in our full model despite the lack of a negative bivariate correlation. This pattern likely reflects a statistical suppression effect, which was confirmed via our formal path analysis, though it should be replicated in future studies before substantive interpretation.

Notably, our findings show that the inverse relationship between resilience and alexithymia was driven by specific cognitive-affective processes. Specifically, EOT was a significant indirect correlate for both conscientiousness and openness. EOT is characterized by a focus on external events and details rather than one’s internal emotional landscape, which can in turn prevent students from engaging in the self-reflection necessary to understand and learn from the stressful experiences of medical education, thereby correlating with lower resilience^49^. This is of particular concern, considering studies among Egyptian medical students have consistently reported low rates of help-seeking behavior, even when experiencing significant stress^17,50,51^. Students displaying high EOT may struggle to articulate their distress or recognize the need for support. Overall, alexithymia was associated with lower resilience, and longitudinal studies are needed to examine whether emotional-processing difficulties affect resilience development.

These specific emotional processing deficits provide a lens for interpreting observed behavioral patterns among medical students in Egypt. For example, one key barrier to mental health support previously identified among medical students in Egypt is a preference to manage problems independently^50^, a pattern closely linked to the negative association we found between DIF, EOT, and resilience, as students who can’t articulate their distress are less likely to seek professional support. Additionally, dissatisfaction with mental health services by Egyptian medical students has also been reported^52^, further discouraging students from seeking much needed support. Given these findings, interventions targeting emotional awareness and expression may be worth evaluating in future resilience-promotion studies. Nevertheless, estimates involving the poorly reliable subscales should be interpreted with caution and require replication using locally validated or psychometrically stronger measures.

Collectively, these findings illustrate the complex nature of resilience, which is associated with sex, academic structure, psychiatric history, emotional regulation, and personality characteristics. From a clinical perspective, it is important to distinguish between stable personality dispositions and modifiable self-regulatory processes; resilience should not be treated merely as a fixed personal trait. Thus, enhancing resilience requires a comprehensive approach. Additionally, sex-specific interventions and targeted support for students with psychiatric conditions or alexithymia may further strengthen resilience-building efforts. By prioritizing these strategies, medical schools can better equip students with the psychological resources necessary to navigate academic and professional challenges effectively.

## Limitations

Certain limitations must be acknowledged when interpreting the findings of our study. For instance, it is important to interpret our findings within the context of our cross-sectional design, which captures resilience as a measurable score at a single point in time and cannot establish causal or developmental relationships. Similarly, the path analysis conducted in this study is exploratory and can only support statistical indirect associations, not temporal mediation or causal mechanisms. However, resilience can also be conceptualized as a dynamic process that evolves throughout a student’s medical education. From the snapshot perspective, our findings suggest static correlations in which traits like neuroticism and alexithymia are associated with a lower resilience score at the time of measurement. Meanwhile, from a dynamic process perspective, the strong negative correlation with neuroticism may not necessarily mean neurotic students are less resilient, but that their tendency toward negative affect may inhibit their ability to develop resilience by impeding adaptation after stressful events^12^. Furthermore, while our study focused on broad personality traits and alexithymia, we did not measure highly modifiable self-regulatory processes, such as worry, rumination, threat and specific coping styles, which are clinically relevant targets for future research.

Additionally, the cutoffs we used for both the CD-RISC-10 and the TAS-20, while used in similar studies among Arabic student populations^34–36^, have not been properly validated for use within this population, which may affect the overall accuracy of the reported categorical estimates. Similarly, certain subscales of the used instruments exhibited low internal consistency (Cronbach’s alpha < 0.70) within our sample, which may affect the precision of the parameter estimates involving these specific constructs and suggests that these translated scales may require further linguistic or cultural adaptation for Egyptian medical populations. Other limitations include the use of convenience sampling, which may introduce selection bias, and the single-center setting, which restricts the generalizability of our findings. Therefore, future research should prioritize longitudinal, multi-center studies utilizing locally validated psychometric tools with representative sampling to provide a more comprehensive understanding of how resilience develops in medical students.

## Conclusion

This study highlights the complex interplay between personality traits, emotional processing (alexithymia), and resilience. Extraversion and conscientiousness were associated with higher resilience, whereas neuroticism, alexithymia, and current psychiatric disorders were linked to lower resilience. The findings further suggest that difficulties in emotional processing may partially explain how certain personality traits are associated with resilience. Collectively, these results emphasize the importance of considering both personality characteristics and emotional awareness in efforts to support medical students’ psychological well-being.

## Supplementary Information

Supplementary Information.

## Data Availability

The data are available from the corresponding author upon request.

## Abbreviations

ANOVA: Analysis of variance
CD-RISC: Connor Davidson resilience scale
ML: Maximum likelihood
MMPME: Mansoura Manchester program for medical education
NEO-FFI: Neuroticism (N), Extraversion (E), Openness (O), Five-factor inventory (FFI)
Q-Q: Quantile–quantile
SD: Standard deviation
SPSS: Statistical package for social science
TAS: Toronto alexithymia scale
VIF: Variance inflation factor
