# Supplementary material for: Exploring the pathways between personality traits, alexithymia, and resilience among medical students and interns: a cross-sectional study in Egypt
Source: Sci Rep. 2026 Jul 2;16:20352. doi: 10.1038/s41598-026-59508-5 (PMC13328675; doi:10.1038/s41598-026-59508-5)
Supplement: Supplementary file 1 — Supplementary Information. [file 41598_2026_59508_MOESM1_ESM.docx]

**Supplementary Table 1.** All direct and indirect effects in the path model predicting resilience among the study’s participants.

| **Pathway** | **Unstandardized Estimate B (95% CI)** | **Standardized β** | **p-value** |
| --- | --- | --- | --- |
| **Direct Effects on Resilience** | | | |
| **Direct Effects on Resilience** |  |  |  |
| Neuroticism → Resilience | -0.24 (-0.31, -0.18) | -0.31 | **< .001** |
| Extraversion → Resilience | 0.15 (0.07, 0.23) | 0.15 | **< .001** |
| Agreeableness → Resilience | -0.10 (-0.17, -0.03) | -0.11 | **0.007** |
| Conscientiousness → Resilience | 0.21 (0.13, 0.29) | 0.24 | **< .001** |
| Openness → Resilience | 0.03 (-0.05, 0.11) | 0.03 | 0.413 |
| Difficulty Describing Feelings (DDF) → Resilience | 0.05 (-0.07, 0.17) | 0.04 | 0.412 |
| Difficulty Identifying Feelings (DIF) → Resilience | -0.10 (-0.20, -0.00) | -0.1 | 0.051 |
| Externally-Oriented Thinking (EOT) → Resilience | -0.16 (-0.26, -0.05) | -0.12 | **0.003** |
| **Indirect Effects on Resilience** |  |  |  |
| Neuroticism → DDF → Resilience | 0.01 (-0.01, 0.03) | 0.01 | 0.429 |
| Neuroticism → DIF → Resilience | -0.03 (-0.07, -0.00) | -0.04 | 0.057 |
| Neuroticism → EOT → Resilience | 0.00 (-0.01, 0.01) | 0 | 0.874 |
| Extraversion → DDF → Resilience | -0.01 (-0.02, 0.01) | -0.01 | 0.434 |
| Extraversion → DIF → Resilience | 0.01 (-0.00, 0.02) | 0.01 | 0.34 |
| Extraversion → EOT → Resilience | 0.01 (-0.00, 0.02) | 0.01 | 0.287 |
| Agreeableness → DDF → Resilience | -0.01 (-0.02, 0.01) | -0.01 | 0.43 |
| Agreeableness → DIF → Resilience | 0.01 (0.00, 0.03) | 0.01 | 0.103 |
| Agreeableness → EOT → Resilience | 0.01 (0.00, 0.02) | 0.01 | 0.071 |
| Conscientiousness → DDF → Resilience | 0.00 (-0.00, 0.01) | 0 | 0.928 |
| Conscientiousness → DIF → Resilience | 0.01 (-0.00, 0.02) | 0.01 | 0.319 |
| Conscientiousness → EOT → Resilience | 0.02 (0.01, 0.03) | 0.02 | **0.019** |
| Openness → DDF → Resilience | -0.00 (-0.01, 0.01) | 0 | 0.853 |
| Openness → DIF → Resilience | -0.00 (-0.01, 0.01) | 0 | 0.919 |
| Openness → EOT → Resilience | 0.04 (0.01, 0.06) | 0.03 | **0.01** |
